# PDIA4 Is a Host Factor Important for Lymphocytic Choriomeningitis Virus Infection

**DOI:** 10.3390/v15122343

**Published:** 2023-11-29

**Authors:** Mengwei Xu, Huan Xu, Weiwei Wan, Xiaoqin Jian, Runming Jin, Lin Wang, Jingshi Wang, Gengfu Xiao, Leike Zhang, Hongbo Chen, Yuxi Wen

**Affiliations:** 1 State Key Laboratory of Virology, Wuhan Institute of Virology, Center for Biosafety Mega-Science, Chinese Academy of Sciences, Wuhan 430071, China; xmw0204@163.com (M.X.); weiwei-wan@outlook.com (W.W.); janelss0520@163.com (X.J.); xiaogf@wh.iov.cn (G.X.); zhangleike@wh.iov.cn (L.Z.); 2 Department of Pediatrics, Union Hospital, Tongji Medical College, Huazhong University of Science and Technology, Wuhan 430022, China; drxu_2020@hust.edu.cn (H.X.); rmjin@hust.edu.cn (R.J.); wl1894@126.com (L.W.); 3 University of Chinese Academy of Sciences, Beijing 100000, China; 4 Department of Hematology, Beijing Friendship Hospital, Capital Medical University, Beijing 100000, China; wangjingshi@ccmu.edu.cn; 5 Hubei Jiangxia Laboratory, Wuhan 430200, China

**Keywords:** lymphoblastic choriomeningitis virus, PDIA4, ATF6, arenavirus

## Abstract

Mammalian arenaviruses are rodent-borne zoonotic viruses, some of which can cause fatal hemorrhagic diseases in humans. The first discovered arenavirus, lymphocytic choriomeningitis virus (LCMV), has a worldwide distribution and can be fatal for transplant recipients. However, no FDA-approved drugs or vaccines are currently available. In this study, using a quantitative proteomic analysis, we identified a variety of host factors that could be needed for LCMV infection, among which we found that protein disulfide isomerase A4 (PDIA4), a downstream factor of endoplasmic reticulum stress (ERS), is important for LCMV infection. Biochemical analysis revealed that LCMV glycoprotein was the main viral component accounting for PDIA4 upregulation. The inhibition of ATF6-mediated ERS could prevent the upregulation of PDIA4 that was stimulated by LCMV infection. We further found that PDIA4 can affect the LCMV viral RNA synthesis processes and release. In summary, we conclude that PDIA4 could be a new target for antiviral drugs against LCMV.

## 1. Introduction

Mammalian arenaviruses are enveloped, ambisense, single-stranded RNA viruses found in close association with specific rodent hosts [1]. Lymphocytic choriomeningitis virus (LCMV), the earliest discovered arenavirus described in the 1930s [2], has a worldwide distribution with a 2 to 5% seroprevalence in humans since its host, *Mus musculus*, can migrate worldwide [3]. LCMV infections are mostly asymptomatic, but they can be fatal in transplant recipients and can cause spontaneous abortion or congenital disorders during fetal development [4]. Other representative members of mammalian arenaviruses such as Lassa virus (LASV), Lujo virus (LUJV), and Machupo (MACV), can cause fatal hemorrhagic disease in humans (case fatality rate up to 35%) [5]. However, there is no FDA-approved antiviral drug or vaccine specific for arenaviruses, except for the broad-spectrum antiviral drug ribavirin. Due to the high homology of arenaviruses, this article aimed to screen and identify new host factors vital for LCMV so as to provide new targets for antiviral drug development for arenaviruses.

The hallmarks of arenaviruses are their insoluble life cycle and their ability to establish sustained infection in cultured cells and in vivo [6]. Since arenaviruses do not integrate into the host genome, their long-term existence requires some degree of sustained replication and the expression of four viral proteins: RNA polymerase (L), matrix protein (Z), nucleocapsid protein (NP), and glycoprotein precursor (GPC) [7]. The life cycle of mammalian arenaviruses can be roughly divided into three stages: entry, replication and transcription, packaging and release. The entry of arenaviruses is mainly mediated by mature GPC (GP1/CP2/SSP complex of the virus). Once entering the cytoplasm, the NP and L are expressed immediately and form the smallest unit for virus transcription and replication together with the viral genome RNA. The matrix protein Z synthesized in the late stage of viral infection can not only regulate the host immune process and inhibit the L-mediated RNA synthesis process but also cooperate with GPC in the packaging and releasing process [7].

This study aimed to identify additional host factors important for LCMV infection that may serve as targets for the development of therapeutics to combat viral infection. Protein disulfide isomerases (PDIs) are well-known multifunctional proteins that are widely involved in numerous diseases, such as infectious diseases, neurodegenerative diseases, metabolic diseases, and cancer. Mammalian PDIs consist of more than 20 proteins [8], which reside mostly in the endoplasmic reticulum (ER) and act as ER chaperones [9]. The ER coordinates the folding and posttranslational maturation of almost all membrane proteins and most secreted proteins, and the accumulation of unfolded or misfolded proteins in the ER results in ER stress (ERS) [10]. ERS is mediated mainly by three specific signal transduction factors: inositol-requiring protein 1 (IRE1), protein kinase RNA(PKR)-like ER kinase (PERK), and activating transcription factor 6 (ATF6) [11]. ATF6 is a 670-amino-acid ER-transmembrane protein that includes two isoforms, ATF6α and ATF6β [12]. Acute LCMV infection is known to selectively induce ATF6-mediated ERS [6]. PDIs were already found as targeting hits in previous mass spectrometry proteomic studies of the LCMV interactome [13].

Recent studies have reported that PDIs reside not only in the ER but also in the nuclei and membrane [14]. This family has been reported to participate in the infection of a variety of viruses, such as influenza virus [15], Ebola virus [16], human astrovirus [17], and human immunodeficiency virus (HIV) [18]. Our present article also found that several PDIs, especially protein disulfide isomerase A4 (PDIA4), were upregulated during LCMV infection. PDIA4 is not essential for the host, since its knockout mice survived without any noticeable phenotype [19]. However, nothing is known about the role of PDIA4 in LCMV infection. We sought to determine whether and how the perturbation of PDIA4 would impede LCMV infection as well as how LCMV infection regulates the expression of PDIA4.

## 2. Materials and Methods

### 2.1. Cell Lines

Human non-small-cell lung cancer cells (A549, ATCC, CCL-185) [20], African green monkey kidney cells (Vero, ATCC, CCL-81), Syrian baby hamster kidney cells (BHK-21, ATCC, CCL-10), and human embryonic kidney SV40 Tag-transformed cells (HEK293T, ATCC, CRL-3216) were obtained from ATCC. The BHK-derived cell line stably expressing T7 RNA polymerase (BSR-T7) was donated by Dr. Mingzhou Chen (Wuhan University, China) and treated with 1 mg/mL G418 (Invitrogen, Carlsbad, California, USA). The above cell lines were routinely tested for the absence of mycoplasma and maintained in high-glucose Dulbecco’s modified eagle’s medium (DMEM, Gibco, Beijing, China) supplemented with 10% (*v*/*v*) fetal bovine serum (FBS, Gibco) at 37 °C in a humidified atmosphere of 5% CO2. Normal human bronchial epithelial (NHBE) cells acquired from the National Collection of Authenticated Cell Culture were stored in our laboratory and maintained in RPMI 1640 (Gibco) supplemented with 10% FBS.

### 2.2. Viruses

Recombinant LCMV expressing LASV or MACV GPC (LCMV-LASV-GPC and LCMV-MACV-GPC viruses) were donated by Dr. Pan Xiaoyan. The LCMV-WT was rescued by using a previously described reverse genetic system [21]. Severe fever with thrombocytopenia syndrome virus (SFTSV) was stored in our laboratory. All experiments involving the viruses were performed in biosafety level 2 (BSL-2) facilities according to the institutional biosafety standard operating procedures.

### 2.3. Plasmids

The coding sequence of each individual LCMV protein (NP, L, GPC, Z) was cloned and inserted into the pCAGGS vector with a C-terminal Twin-Strep-tag (TS). The cDNA sequence of human PDIA4 was cloned and inserted into the pLX304 vector with a V5 tag. pCAGGS-EGFP-TS, pT7 plasmids encoding L and S segments of LCMV (pT7-LCMV-S, pT7-LCMV-L) were obtained from Dr. Wan Weiwei [22]. All the above plasmids were validated by Sanger DNA sequencing prior to use.

### 2.4. Antibodies

Rabbit anti-LCMV-NP serum was generated by immunizing rabbits with purified viral NP proteins [22]. The anti-Strep mouse monoclonal antibody (AE066), horseradish peroxidase (HRP)-conjugated GAPDH (glyceraldehyde-3-phosphate dehydrogenase) antibody (AC035), and anti-PDIA6 rabbit polyclonal antibody (A18092) were purchased from ABclonal (Wuhan, China). The anti-PDIA3 rabbit polyclonal antibody (15967-1-AP), anti-PDIA4 rabbit polyclonal antibody (14712-1-AP), anti-PDIA5 rabbit polyclonal antibody (15545-1-AP), anti-V5 antibody (14440-1-AP), horseradish peroxidase (HRP)-conjugated goat anti-rabbit IgG(H+L) (SA00001-2), and HRP-conjugated goat anti-mouse IgG(H+L) (SA00001-1) were purchased from Proteintech Group (Wuhan, China).

### 2.5. Quantitative Proteomics Analysis

The samples were lysed with fresh lysate buffer (1% SDC/100 mM Tris-HCl, pH 8.5/10 mM TCEP/40 mM CAA), which was followed by inactivation in a water bath at 60 °C for 30 min, denaturation, reduction, and alkylation. The protein that gathered in the supernatant following centrifugation was then incubated with trypsin (protein:enzyme = 50:1) at 37 °C overnight for enzyme digestion. On the second day, the peptides were desalted using a column, drained by a centrifugal concentrator, and frozen at −20 °C.

Equivalent samples were taken for TMT labeling according to the TMT manufacturer. The labeled samples were mixed and desalted using Sep-Pak C18. After vacuum draining, the mixed samples were separated by high-pH reverse chromatography and finally combined into 15 components. After vacuuming, the samples were stored in a −80 °C refrigerator for further testing. 

Mass spectrometry was performed using Thermo’s Orbitrap Exploris 480 LMS system. The samples were separated by a liquid-phase EASY-nLC 1200 system with nanoliter flow rates. The peptide samples were dissolved in loading buffer, inhaled by an automatic injector, and separated by an analysis column (2 μm, 120 A, 75 μm × 250 mm). The analytical gradient was established using two mobile phases (mobile phase A: 0.1% formic acid and mobile phase B: 0.1% formic acid, 90% ACN). The liquid phase flow rate was set to 300 nL/min. For mass spectrometry DDA mode analysis, one MS full scan (R = 60 K, AGC = standard, max IT = 25 ms, scan range = 350–1500 m/z) was included in each scan cycle. In addition, several subsequent MS/MS scans (R = 15 K, AGC = standard, max IT = 22 ms) were performed using the TurboTMT mode, and the cycle time was set to 2 s. The HCD collision energy was set to 36. The isolation window of the parent ion was set to 1.2 Da. The dynamic exclusion time of the repeated ion collection was set to 35 s.

The mass spectrum data were retrieved by the MaxQuant (V1.6.6) software, and the database retrieval algorithm was Andromeda. The mouse proteome reference database in UniProt was used for searching. The search results were screened based on the 1% FDR level of proteins and peptides, and the entries of anti-library proteins, polluting proteins, and proteins with only one modified peptide segment were deleted. The remaining identification information was used for subsequent analysis.

### 2.6. Western Blot

The supernatants were removed, and the cells were washed with PBS three times. Then, the cells were lysed by radioimmunoprecipitation assay (RIPA) lysis buffer (Beyotime, Shanghai, China) supplemented with 5× SDS-PAGE sample loading buffer and heated at 95 °C for 10 min. Then, the proteins were separated on SDS-PAGE gels and transferred onto polyvinylidene difluoride membranes by a Trans-Blot Turbo rapid transfer system (Bio-Rad, Hercules, CA, USA) according to the manufacturer’s instructions. The membranes were blocked in Tris-buffered saline (TBS) supplemented with 5% milk for 2 h at room temperature and then incubated with a primary antibody at 4 °C overnight. The membranes were then washed in TBS supplemented with 0.1% Tween 20 and incubated with the HRP-conjugated secondary antibody for 1 h at room temperature. The membranes were washed and imaged.

### 2.7. RNAi Experiments

The targeting siRNAs used in this study were purchased from GenePharma (Shanghai, China). siRNAs were transfected into cells using Lipofectamine RNAiMAX transfection reagent (Thermo Fisher, San Jose, CA, USA) according to the manufacturer’s protocol. siRNA without human mRNA targets (NC) was used as a control for RNAi-related experiments. All siRNA sequences are listed in Table S2.

### 2.8. Construction of Scramble-/PDIA4-/PDIA5-Knockout (KO) A549 Cells

sgRNA sequences targeting exons of human PDIA4 and PDIA5 were designed by using the Millipore & Sigma CRISPR design tool (https://www.milliporesigmabioinfo.com/bioinfo_tools/faces/informatics.xhtml, accessed on 23 June 2021). sgRNA oligos were synthesized, annealed, and ligated to a FastDigest BsmBI (Thermo Fisher, USA)-digested lentiCRISPR v2 plasmid by using T4 DNA ligase (Thermo-Fisher, USA). The LentiCRISPR v2 plasmid containing sgRNA with no human genome target sequence (Scramble) was used as a control. The lentiviruses were harvested from supernatants of HEK293T cells transfected with Scramble-/PDIA4-/PDIA5-sgRNA LentiCRISPR v2 plasmid (1000 ng) and two package plasmids (pCMV-dR8.91 (1500 ng) encoding HIV-Gag and HIV-Pol proteins and pMD2.G (500 ng) encoding VSV-G proteins) by Liopfectamine 2000 at 48 h post transfection. The supernatants were filtered through a 0.22 μm PES filter, and the A549 cells were infected three times. Then, the infected A549 cells were incubated with 0.5 μg/mL puromycin for another 5 days and serially diluted for monoclonal cells without puromycin. All sgRNA sequences are listed in Table S2.

### 2.9. Mouse Experiment

Six- to ten-week-old C57BL/6-Prf1^tm1Sdz^/J mice were infected with 2 × 10^5^ P.F.U. of LCMV-Armstrong. At 5 days post infection (d.p.i.), the mice were euthanized, and the lung and kidney were dissected and homogenized for RT-PCR.

### 2.10. Statistical Analysis

Data from cell and animal studies were analyzed using the Prism 9.0 software and reported as the mean±SD. The statistical significance was calculated using unpaired *t* test analyses or one-way ANOVA analyses. Significance was set to *p* < 0.05 (* *p* < 0.05, ** *p* < 0.01, *** *p* < 0.001, **** *p* < 0.0001).

## 3. Results

### 3.1. Identification of New Players in LCMV Infection through a Quantitative Proteomic Screen

**Figure 1:**
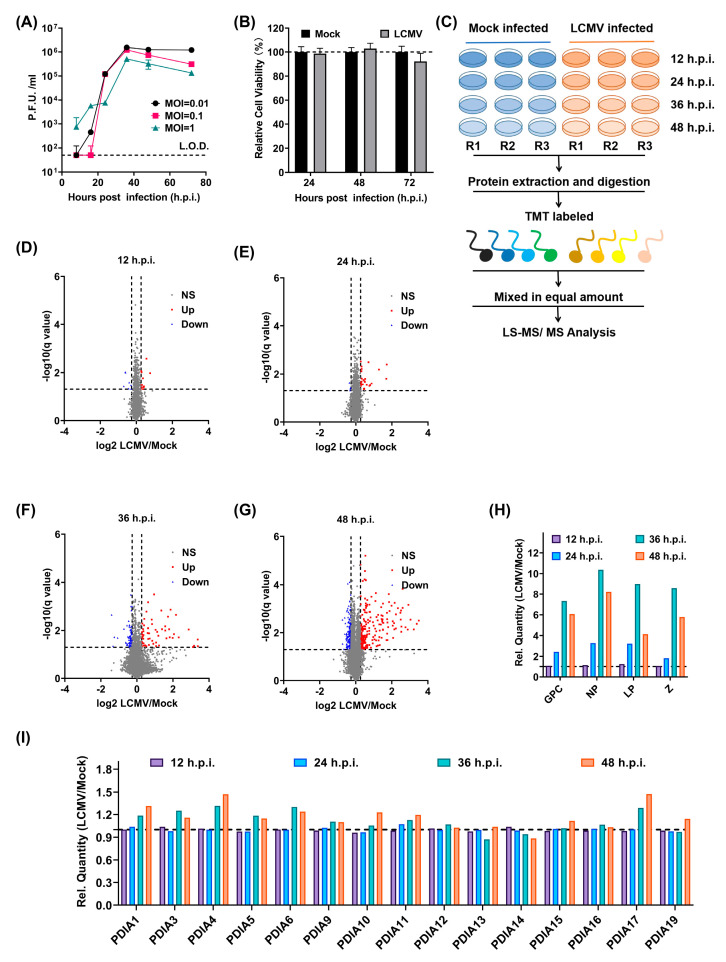
Screening host factors important for LCMV infection using quantitative proteomic analysis. (**A**) The growth curve of LCMV in A549 cells at MOI of 0.01, 0.1, and 1. A549 cells were plated at a density of 3 × 10^5^/mL and were infected with LCMV at MOI of 0.01, 0.1, and 1; at 8, 16, 24, 36, 48 and 72 h.p.i., the supernatants were gathered and quantified by immune plaque assay. (**B**) The viability of A549 cells infected by LCMV at an MOI of 0.01 determined by CCK8 (C0038, Beyotime) according to the instructions at 24, 48, and 72 h.p.i. (**C**) The schematic representation of the quantitative proteomics analysis. (**D**–**G**) The volcanic map for detectable proteins at 12, 24, 36, and 48 h.p.i. (**H**) The relative protein quantity of four viral proteins determined by quantitative proteomics analysis at 12, 24, 36, and 48 h.p.i. relative to 12 h.p.i. (**I**) The relative protein expression level of detectable PDIs in A549 cells determined by quantitative proteomic analysis at 12, 24, 36, and 48 h.p.i. relative to mock–infected A549 cells.

To screen new host factors important for LCMV infections, we first determined the appropriate infection time points as well as the viral inoculum for further study in the human lung epithelial cell Line A549, which is known to be susceptible to LCMV [23]. The viral titers in the supernatants collected at serial time points after infection with different multiplicities showed that LCMV production reached it plateau early before 48 h.p.i. LCMV-infected A549 cells at a multiplicity of infection (MOI) of 0.01 amplified the best (Figure 1A). We further confirmed that the infection of A549 cells by LCMV at an MOI of 0.01 had nearly no effect on cell viability (Figure 1B). Once the appropriate time and dose were determined, we carried out the quantitative proteomics analysis following the scheme shown in Figure 1C. Up to 8364 host factors in the mock-/LCMV-infected A549 cell lysis were identified by quantitative proteomics at four time points (12, 24, 36, 48 h.p.i.) (Table S1). The numbers of differentially expressed proteins at 12, 24, 36, and 48 h.p.i. were 18, 26, 119, and 365, respectively (Figure 1D–G). The relative expression levels of the four LCMV proteins peaked at 36 h.p.i. (Figure 1H). After careful comparison and analysis, we found that a large variety of ERS-related proteins were upregulated, including 8 out of 15 detectable protein disulfate isomerases (PDIs). PDIs were differentially expressed and reached their peak at 48 h.p.i., a litter later than the replication peak of LCMV (Figure 1I).

### 3.2. LCMV Infection Promotes the Expression of PDIs, Especially PDIA4, Both In Vitro and In Vivo

**Figure 2:**
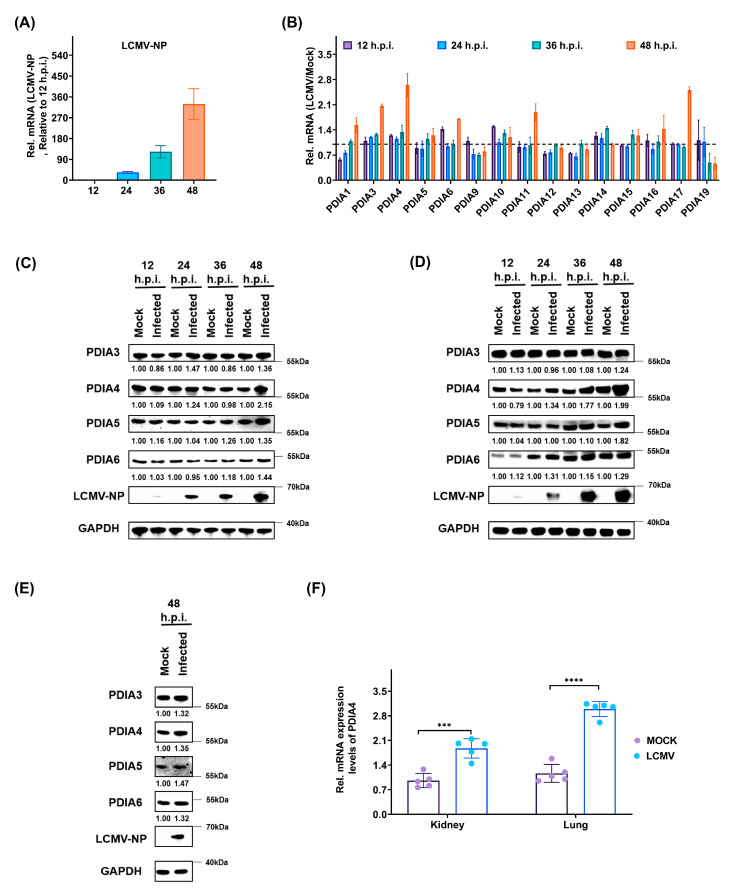
The PDIA4 expression level is upregulated by LCMV infection both in vitro and in vivo. (**A**) The expression level of LCMV-NP in A549 cells at 12, 24, 36, and 48 h.p.i. determined by RT-PCR. (**B**) The expression level of detectable PDIs in A549 cells at 12, 24, 36, and 48 h.p.i. determined by RT-PCR. (**C**) The protein expression level of PDIA3, PDIA4, PDIA5, and PDIA6 in mock-/LCMV-infected A549 cells at 12, 24, 36, and 48 h.p.i. determined by Western blot. (**D**) The protein expression level of PDIA3, PDIA4, PDIA5, and PDIA6 in mock-/LCMV-infected HEK293T cells at 12, 24, 36, and 48 h.p.i. determined by Western blot. (**E**) The protein expression level of PDIA3, PDIA4, PDIA5, and PDIA6 in mock-/LCMV-infected NHBE cells at 48 h.p.i. determined by Western blot. (**F**) The relative mRNA expression level of PDIA4 in the kidney and lung of mock-/LCMV-infected mice (relative to mock, n = 5, unpaired *t* test). *** *p* < 0.001, **** *p* < 0.0001.

To further confirm the upregulation of PDIs during LCMV infection, we first detected the expression level of PDIs in A549 cells using RT-PCR. The expression level of LCMV-NP was detected to confirm effective LCMV infection (Figure 2A). Then, the relative transcription levels of 15 detectable PDIs in the LCMV-infected samples were compared to those in the mock-infected samples. We found that some PDIs were upregulated (especially PDIA4), which was consistent with the results of the quantitative proteomic analysis (Figure 2B). The protein expression levels of PDIA3, PDIA4, PDIA5, and PDIA6 in the mock-/LCMV-infected A549 cells at 12, 24, 36, and 48 h.p.i. were verified by Western blotting (Figure 2C). Then, we also examined whether the expression level of PDIA4 would be regulated by LCMV infection in other permissive cells. Using Western blotting, we determined the expression levels of PDIA3, PDIA4, PDIA5, and PDIA6 in mock-/LCMV-infected HEK293T cells at 12, 24, 36, and 48 h.p.i. (Figure 2D) and in human bronchial epithelial NHBE cells at 48 h.p.i. (Figure 2E). We found that LCMV infection promoted the protein expression levels of PDIA3, PDIA4, PDIA5, and PDIA6 at 48 h.p.i. in these two permissive LCMV cell lines, and the PDIA4 and PDIA5 upregulation results were more convincing than those of PDIA3 and PDIA6. In addition, we also detected the expression level of PDIA4 in vivo (Figure 2F). Briefly, kidney and lung, the two reported permissive tissues for LCMV infection [21], were obtained from either the LCMV-infected or mock-treated mice, and the mRNA expression level of PDIA4 was examined. As shown in Figure 2F, the mRNA expression levels of PDIA4 in the kidney and lung were higher in the LCMV-infected mice, suggesting that LCMV can upregulate PDIA4 expression in vivo.

### 3.3. PDIA4 Is a Positive Regulator in LCMV Infection

**Figure 3:**
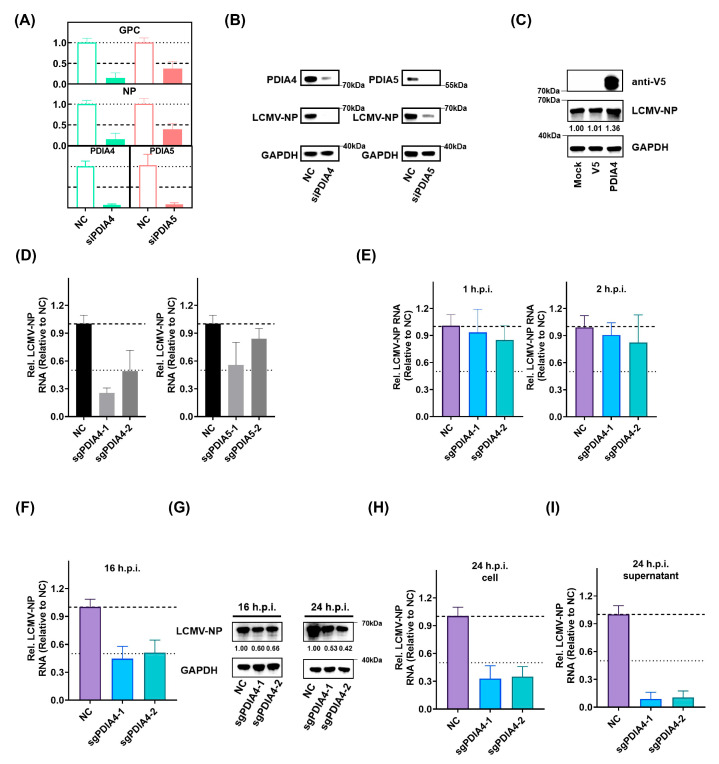
The upregulation of PDIA4 promotes LCMV infection. (**A**) The relative RNA expression levels of LCMV GPC and NP in LCMV-infected A549 cells that were pretreated with siRNAs targeting PDIA4/PDIA5 or the NC siRNA for 48 h at 36 h.p.i. (**B**) The protein level of LCMV NP in LCMV-infected A549 cells that were pretreated with siRNAs targeting PDIA4/PDIA5 or the NC siRNA for 48 h at 36 h.p.i. (**C**) The protein level of LCMV NP in LCMV-infected A549 cells that were pretreated with mock/pLX304-V5/pLX304-PDIA4-V5 plasmids for 48 h at 36 h.p.i. (**D**) The relative RNA expression level of LCMV-NP in LCMV-infected Scramble-KO (NC), PDIA4-KO, and PDIA5-KO A549 cell lysates at 48 h.p.i. (**E**) The relative RNA expression level of LCMV-NP in LCMV-infected Scramble-KO (NC), PDIA4-KO, and PDIA5-KO A549 cell lysates at 1 h.p.i. and 2 h.p.i. (**F**) The relative RNA expression level of LCMV-NPs in LCMV-infected Scramble-KO (NC), PDIA4-KO, and PDIA5-KO A549 cells lysates at 16 h.p.i. (**G**) The protein expression level of LCMV-NPs in LCMV-infected Scramble-KO (NC), PDIA4-KO, and PDIA5-KO A549 cells lysates at 16 h.p.i. and 24 h.p.i. (**H**) The relative RNA expression level of LCMV-NPs in LCMV-infected Scramble-KO (NC), PDIA4-KO, and PDIA5-KO A549 cell lysates at 24 h.p.i. (**I**) The relative RNA expression level of LCMV-NPs in LCMV-infected Scramble-KO (NC), PDIA4-KO, and PDIA5-KO A549 supernatants at 24 h.p.i.

To validate the roles of PDIs in LCMV infection, we separately inactivated PDIA4 and PDIA5 in A549 cells by transient transfection with targeting siRNAs, which led to the conclusion that the LCMV viral RNA synthesis level was suppressed as the expression of PDIA4 and PDIA5 was inhibited (Figure 3A,B). In addition, we expressed PDIA4 in A549 cells exogenously (Figure 3C) and found that the addition of exogenous PDIA4 also promoted LCMV replication. To further determine the specific step by which PDIA4 and PDIA5 influence LCMV infection, we constructed PDIA4- (PDIA4-KO) and PDIA5-knockout (PDIA5-KO) A549 cells using CRISPR-Cas9 gene editing technology. We confirmed that the respective genes were inactivated in the PDIA4- and PDIA5-KO cells by Western-blot analysis (Figure S1). Each of the cell types was infected with LCMV, and the extent of infection was determined using RT-PCR at 24 h.p.i. In line with the PDIA4 and PDIA5 siRNA experiments, infection with LCMV showed a reproducible reduction in PDIA4-KO A549 cells and a slight reduction in PDIA5-KO A549 cells (Figure 3D). The life cycle of LCMV infection can be divided into three stages: entry, transcription and replication, and release. We set out to determine which stage PDIA4 mainly participates in by detecting the LCMV-NP expression level using qPCR at serial time points. As shown in Figure 3E, the knockout of PDIA4 seemed to have no effect on LCMV viral RNA synthesis level at 1 and 2 h.p.i., suggesting that PDIA4 did not affect the entry of LCMV. At 16 h.p.i., the LCMV was just starting the first round of release, as shown in Figure 1A; thus, we analyzed the replication capacity of LCMV at 16 h.p.i. by detecting the LCMV viral RNA synthesis level in cell lysates (Figure 3F,G). The viral RNA synthesis level of LCMV at 16 h.p.i. was obviously inhibited in the PDIA4-KO A549 cells compared with that in the Scramble-KO A549 cells (negative control, NC), suggesting that the knockout of PDIA4 inhibits the viral RNA synthesis level of LCMV. In addition, we chose 24 h.p.i., which was not enough for the second round of release, to be the appropriate time point for analyzing the release capacity of LCMV by detecting LCMV in supernatants (Figure 3G–I). The reduction in the viral RNA levels in the viral particles present in the supernatants of PDIA4-KO A549 cells was more remarkable than that in the cell lysate compared with that in the Scramble-KO A549 cells, suggesting that the knockout of PDIA4 inhibited the release of LCMV. As shown in Figure S2, we tested the effect of PDIA4-KO in cells transfected with Z coding plasmid and found no significant change. The interactions between Z and GPC determine the final outcome of virus release, and we speculated that PDIA4 may affect virus release mainly by interacting with GPC but not Z. Collectively, these data directly demonstrate a positive role of PDIA4 in LCMV infection.

### 3.4. ATF6-Mediated PDIA4 Inhibition Suppresses LCMV Infection

**Figure 4:**
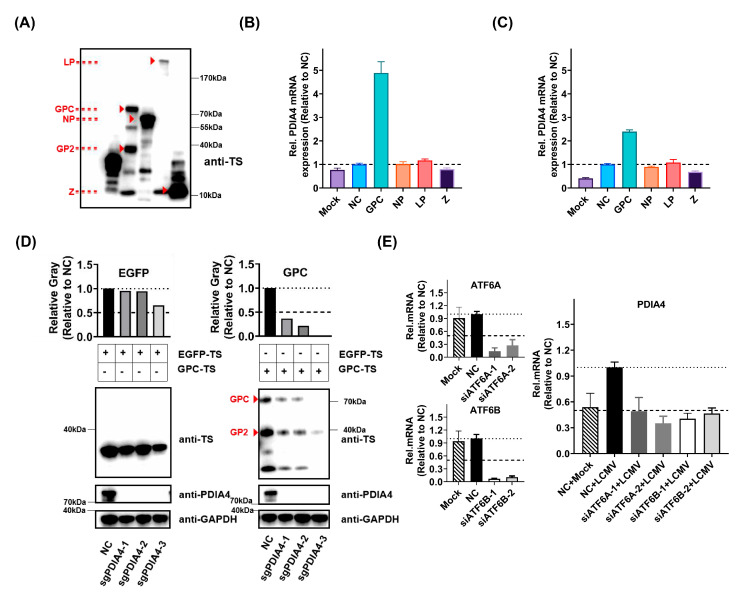
LCMV infection promotes PDIA4 expression by activating ATF6−mediated ERS. (**A**) The expression of LCMV GPC, NP, L, and Z in HEK293T cells by transfection of pCAGGS−LCMV−GPC−TS, pCAGGS−LCMV−NP−TS, pCAGGS−LCMV−L−TS, and pCAGGS−LCMV−Z−TS plasmids separately. (**B**) The relative mRNA expression level of PDIA4 in mock−, pCAGGS−TS−, pCAGGS−LCMV−GPC−TS−, pCAGGS−LCMV−NP−TS−, pCAGGS−LCMV−L−TS−, and pCAGGS−LCMV−Z−TS−transfected A549 cells at 36 h post transfection determined by RT-PCR. (**C**) The relative mRNA expression level of PDIA4 in mock−, pCAGGS−TS−, pCAGGS−LCMV−GPC−TS−, pCAGGS−LCMV−NP−TS−, pCAGGS−LCMV−L−TS−, and pCAGGS−LCMV−Z−TS−transfected HEK293T cells at 36 h post transfection determined by RT-PCR. (**D**) The protein expression level of EGFP and LCMV−GPC in Scramble−/PDIA4−KO A549 cells transfected with pCAGGS−EGFP−TS and pCAGGS−LCMV−GPC−TS plasmids. (**E**) The relative mRNA expression level of PDIA4 in mock−/LCMV−infected A549 cells pretreated with siRNAs targeting ATF6A and ATF6B for 48 h at 36 h.p.i. determined by RT−PCR.

To further determine the interactions between LCMV and PDIA4, we successfully constructed and expressed the four components of LCMV (GPC, NP, L, Z) in A549 and HEK293T cells (Figure 4A). We found that GPC was the key factor that promoted the expression of PDIA4 in both the A549 and HEK293T cells (Figure 4B,C). The expression level of GPC in the PDIA4-KO cells was obviously blocked compared to that in wild-type A549 cells, while there was nearly no difference in the expression level of EGFP (Figure 4D). It has been reported that ERS mediates the activation of PDIA4 [18], and acute LCMV was reported to selectively induce ATF6-mediated ERS. We then set out to determine whether the regulation of ATF6 would affect the intracellular level of PDIA4. By knocking down ATF6A and ATF6B expression levels in A549 cells, we found that LCMV infection failed to promote PDIA4 expression (Figure 4E).

**Figure 5:**
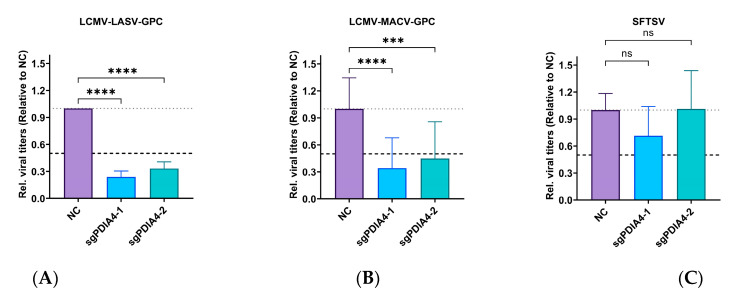
PDIA4 is important for other arenavirus infections. (**A**) The relative viral titers of supernatants gathered from LCMV-LASV-infected Scramble (NC)-/PDIA4-KO A549 cells at 24 h.p.i. (**B**) The relative viral titers of supernatants gathered from LCMV-MACV-infected Scramble (NC)-/PDIA4-KO A549 cells at 24 h.p.i. (**C**) The relative viral titers of supernatants gathered from SFTSV-infected Scramble (NC)-/PDIA4-KO A549 cells at 48 h.p.i. (relative to NC, one-way ANOVA test). *** *p* < 0.001, **** *p* < 0.0001, and ns = no significant.

To further determine whether PDIA4 would be a potential antiviral target for other arenaviruses, we also involved a panel of LCMV-arenavirus chimeras. By detecting the viral titers of LCMV-MACV and LCMV-LASV in the supernatants of LCMV-MACV- or LCMV-LASV-infected PDIA4-KO A549 cells at 24 h.p.i., we verified that the genetic ablation of PDIA4 would affect the infection of LCMV-MACV and LCMV-LASV (Figure 5). In addition, we found that the infection level of SFTSV, which is a bunyavirus, was not affected.

## 4. Discussion

LCMV is an obligate intracellular pathogen similar to other viruses, which must take advantage of the host to enter cells, evade the host immune system, and transform the cellular environment into one that is conductive to viral propagation [24]. Virus–host interactions are numerous and dynamic. Various cellular processes are hijacked during the viral infection, and the same pathway could be regulated in opposite directions at different stages of the viral life cycle. It is necessary for us to use scientific techniques that are able to observe dynamic changes in such processes over a time course of LCMV infection. The study of virus–host interactions through a quantitative proteomics strategy is an important scientific weapon in the fight against viral infection.

Our present article first described the numerous and dynamic interactions between LCMV and the host using quantitative proteomics analysis, and more than 8000 host proteins were quantified, among which 18, 26, 119, and 365 kinds of proteins were differentially regulated at 12, 24, 36, and 48 h.p.i., respectively. We believe that further study on these proteins may lead to the discovery of important factors for LCMV replication and provide antiviral targets for arenaviruses. The synthesis and proper folding of most viral glycoproteins are accomplished on the ER and depend on numerous molecular chaperones. The main ER chaperones are the glucose-regulated chaperones GRP78 and GRP94, the carbohydrate-like or lectin-like chaperones calnexin (CNX) and calreticulin (CRT), PDIs, and DNAJ chaperones [25]. The expression levels of a large variety of ER proteins, such as HSPA5, ERAP1, PDIA4, and AGR2, were upregulated concomitantly with the expression peak of LCMV GPC in our quantitative proteomic analysis, as shown in Supplementary Table S1.

In addition to describing the dynamic viral–host interaction, our article also aimed to screen for new host factors vital for LCMV infection and even other mammalian arenaviruses. We found that several kinds of PDIs (especially PDIA4) were upregulated during LCMV infection in A549 cells. Then, we also proved that the upregulation of PDIA4 was universal in other LCMV-infected cells and LCMV-infected mice. We then set out to clarify whether and how the upregulated PDIA4 facilitates LCMV infection. Using the function gain or loss method, PDIA4 was convincingly proven to be a host factor that facilitates the infection of LCMV in host cells. We also declared that PDIA4 influenced the viral RNA synthesis level of LCMV infection as well as the release processes of LCMV infection. However, further experiments are needed for reaffirming the role of PDIA4 in viral release.

The expression of individual viral proteins (GPC, NP, L, Z) revealed that the promotion of PDIA4 was mainly caused by viral GPC but not by the other viral proteins. Studies have reported that the expression of structural proteins of the viral envelope in the ER could be a trigger for ERS once the folding capacity is overwhelmed, resulting in the accumulation of unfolded proteins [10]. PDIA4 is predominantly located in the ER [9]. Previous studies have shown that the expression of PDIA4 is a self-protective response induced by ERS, which can mediate oxidative protein folding, inhibit apoptosis, and promote tumor progression by combining and degrading misfolded proteins [18]. Mechanistically, PDIA4 is involved in the PI3K/AKT/m-TOR and PERK/ATF4 pathways [26,27]. We reasonably hypothesized that unfolded GPC accumulates in the ER during LCMV infection and promotes PDIA4 expression in the ER by selectively inducing ATF6-mediated ERS. As a downstream effector of ATF6-mediated ERS, upregulated PDIA4 promotes the proper folding of LCMV GPC to facilitate the biosynthesis of large amounts of viral GPC needed for optimal virus production during acute infection and protect the cell against virus-inflicted damage. We further proved the requirement of ATF6-mediated signaling in the promotion of PDIA4 during LCMV infection. In addition, the inhibition of PDIA4 also influenced the infection of a panel of LCMV-arenavirus chimeras. 

Overall, this study unraveled the important role of PDIA4 in LCMV viral RNA synthesis and release. ATF6-mediated ERS is involved in the promotion of PDIA4 during LCMV infection. The inhibition of ATF6 resulted in a reduction in the LCMV infection. In addition, our study adds to our understanding of viral–host interactions and may shed new light on future antiviral drug studies of mammalian arenaviruses.

## Data Availability

Data are contained within the article and Supplementary Materials.

## Supplementary Materials

The following supporting information can be downloaded at: https://www.mdpi.com/article/10.3390/v15122343/s1. Figure S1: The monoclonal cell screening of PDIA4- and PDIA5-KO A549 cells; Figure S2. The budding assay; Table S1: The sequences of primers, siRNAs, and sgRNAs; Table S2: The data of quantitative proteomics for mock-/LCMV-infected A549 cells.
