# Supplementary material for: PDIA4 Is a Host Factor Important for Lymphocytic Choriomeningitis Virus Infection
Source: Viruses. 2023 Nov 29;15(12):2343. doi: 10.3390/v15122343 (PMC10747894; doi:10.3390/v15122343)
Supplement: Supplementary file 1 [file viruses-15-02343-s001.zip › Supplementary Figure.pdf]

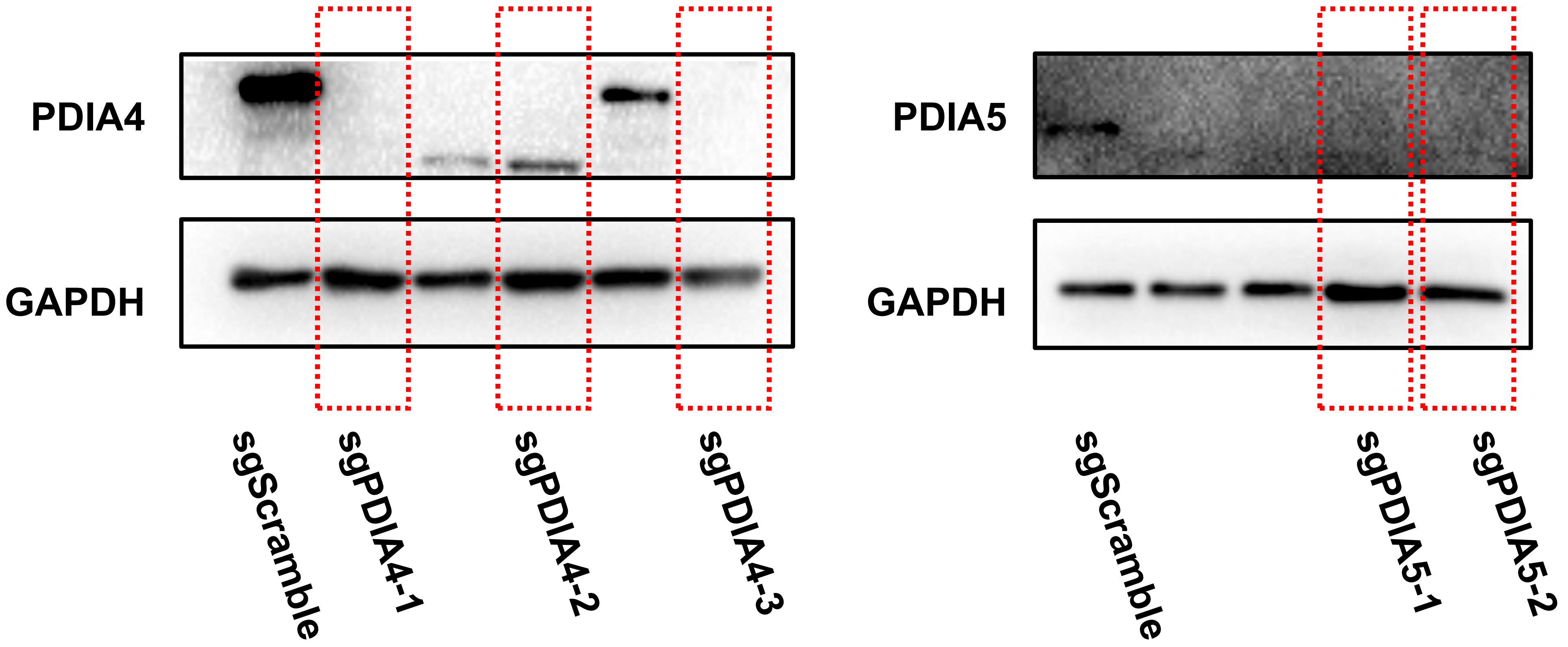

Supplementary Figure S1. The monoclonal cell screening. The monoclonal cells were preseeded in 12 well-plate, and the cells were washed and lysed for Western blot after 24 hours. The indicated cells with red rectangular box were chosed for follow-up experiment

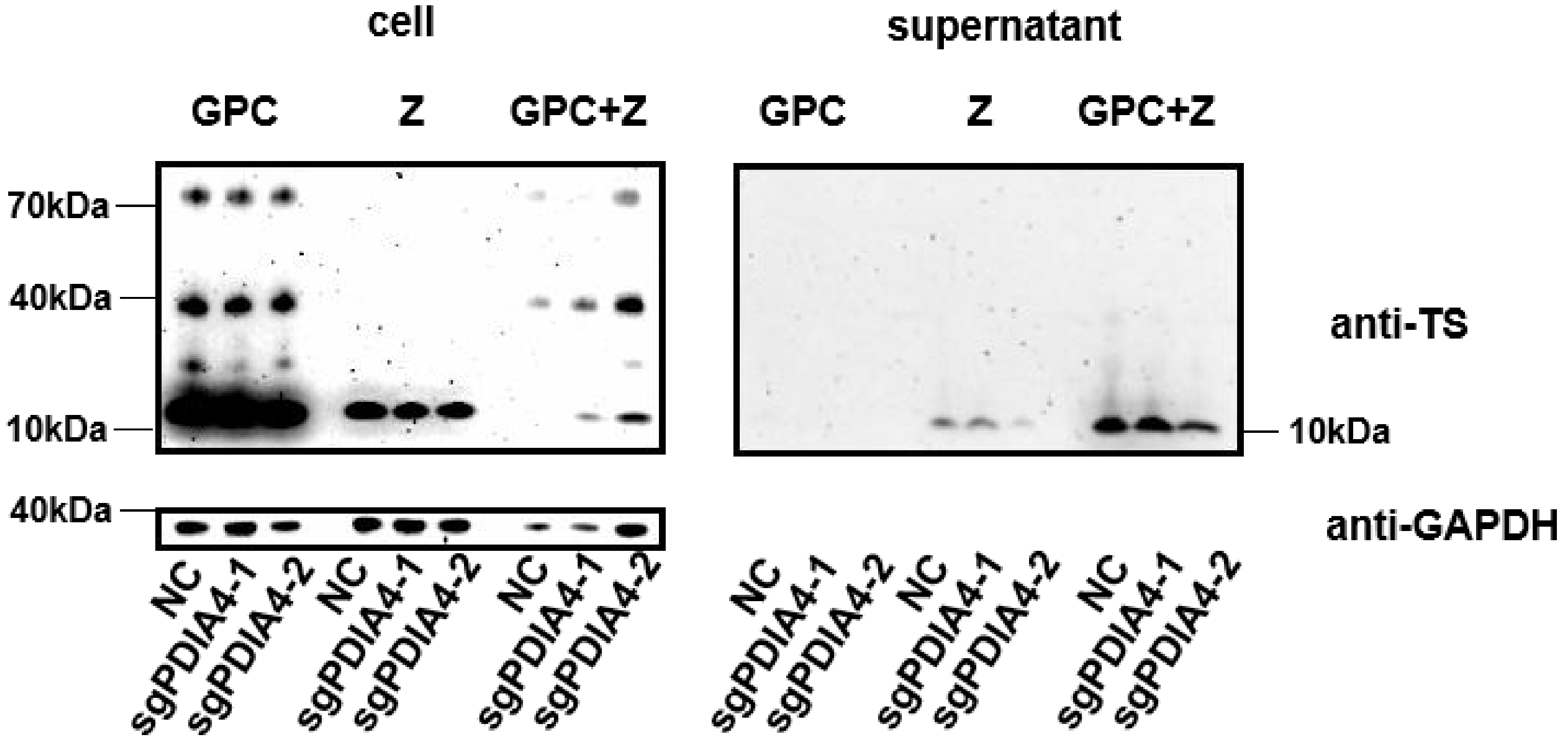

Supplementary Figure S2. The budding assay. The scramble-KO (NC), PDIA4-KO cells were preseeded in 12 well-plate, then the cells were transfected with pCAGGS-LCMV-GPC-TS, pCAGGS-LCMV-Z-TS separately and co-transfected with pCAGGS-LCMV-GPC-TS and pCAGGS-LCMV-Z-TS, cells and supernatants were gathered for Western blot after 48 hours.
