# Supplementary material for: PDIA4 Is a Host Factor Important for Lymphocytic Choriomeningitis Virus Infection
Source: Viruses. 2023 Nov 29;15(12):2343. doi: 10.3390/v15122343 (PMC10747894; doi:10.3390/v15122343)
Supplement: Supplementary file 1 [file viruses-15-02343-s001.zip › supplementary Table S2.pdf]

Supplementary Table S2. The sequence of primers for RT-PCR, siRNAs and sgRNAs

**Primers**	
LCMV-NP F	GTACAAGCGCTCACAGACCT
LCMV-NP R	GTTACCCCCATCCAACAGGG

**siRNAs**	
**Target**	**sequence**
siATF6A-1	GCAGCAACCAATTATCAGTTT
siATF6A-2	GACACATCAGATGGTATTATC
siATF6B-1	CCAGCATTCTTGGATGCAATT
siATF6B-2	CTCTCCATGTTCTTCCGTCAA
siPDIA4	CCGCAAAGGAAGGCCUUAUTT
siPDIA5	GCAGAAAGAUUCCACAUCUTT
**sgRNAs**	
**Target**	**sequence**
sgPDIA4-F	CACCGGCGCTGAGGTTGCATCGATCT
sgPDIA4-R	AAACAGATCGATGCAACCTCAGCGC
sgPDIA5-F	CACCGGATTGCCAGCAGCAGCCACGC
sgPDIA5-R	AAACGCGTGGCTGCTGCTGGCAATC
